# Fluorine-Terminated Polycrystalline Diamond Solution-Gate Field-Effect Transistor Sensor with Smaller Amount of Unexpectedly Generated Fluorocarbon Film Fabricated by Fluorine Gas Treatment

**DOI:** 10.3390/ma15092966

**Published:** 2022-04-19

**Authors:** Yukihiro Shintani, Hiroshi Kawarada

**Affiliations:** 1 Graduate School of Science and Engineering, Waseda University, 3-4-1 Okubo, Shinjuku, Tokyo 169-8555, Japan; kawarada@waseda.jp; 2 Department of Management Information Sciences, Faculty of Social Systems Science, Chiba Institute of Technology, 2-17-1 Tsudanuma, Narashino, Chiba 275-0016, Japan; 3 The Kagami Memorial Laboratory for Materials Science and Technology, Waseda University, 2-8-26 Nishiwaseda, Shinjuku, Tokyo 169-0051, Japan

**Keywords:** polycrystalline diamond, fluorine gas treatment, boron-doped diamond, fluorine-termination, electrolyte-solution-gate field-effect transistor

## Abstract

In this study, a partially fluorine-terminated solution-gate field-effect transistor sensor with a smaller amount of unexpectedly generated fluorohydrocarbon film on a polycrystalline diamond channel is described. A conventional method utilizing inductively coupled plasma with fluorocarbon gas leads the hydrogen-terminated diamond to transfer to a partially fluorine-terminated diamond (C–F diamond); an unexpected fluorohydrocarbon film is formed on the surface of the diamond. To overcome this issue, we newly applied fluorine gas for the fluoridation of the diamond. Analytical results of X-ray photoelectron spectroscopy and time-of-flight secondary ion mass spectrometry suggest that the fluorocarbon film does not exist or only a smaller amount of fluorocarbon film exists on the diamond surface. Conversely, the C–F diamond fabricated by the conventional method of inductively coupled plasma with a perfluoropropane gas (C_3_F_8_ gas) source possesses a certain amount of fluorocarbon film on its surface. The C–F diamond with a smaller amount of unexpectedly generated fluorohydrocarbon film possesses nearly ideal drain–source–voltage vs. gate–source–current characteristics, corresponding to metal–oxide–silicon semiconductor field-effect transistor theory. The results indicate that the fluorine gas (F_2_ gas) treatment proposed in this study effectively fabricates a C–F diamond sensor without unexpected semiconductor damage.

## 1. Introduction

The surface modification of diamond is an effective means of controlling its physical/chemical properties, i.e., biological affinity, friction, electrical/electrochemical properties, wettability [1]. Plasma treatment is one of the most efficient methods for the introduction of a heteroatom onto the surface of a diamond within a shorter time, including a chlorine termination (C–Cl termination) using chloride gas (Cl_2_ gas) [2], an oxygen termination (C–O termination) using oxygen gas (O_2_ gas) [3,4], and a hydrogen termination (C–H termination) using hydrogen gas (H_2_ gas) [5,6,7], respectively. The surface of a fluorine-terminated diamond (C–F diamond) is attractive because of its unique properties, i.e., low coefficient of friction, hydrophobicity, and chemical/biochemical inertness [8,9,10,11,12,13,14]. In addition, the diamond fluorination increases the overpotential of hydrogen evolution reactions in electrolyte aqueous solutions, where it could be an advantage for sensor use [14,15].

Authors and our group successfully induced diamond semiconductors for a no-gate-insulator SGFET (electrolyte-solution-gate field-effect transistor) utilizing a single-crystal diamond [16,17,18,19], a polycrystalline diamond [12,20,21], and a boron-doped diamond [12,20,22]. An electric double layer on the diamond surface leads to its operation as a gate insulator in these devices [16]. A partially oxygen-terminated diamond (C–O diamond) and partially nitrogen-terminated diamond (C–N diamond) have also been successfully induced to control ion sensitivity for sensors [22]. Since its specific properties, such as hydrophobicity, the C–F diamond surface has also become a focus of attention for the use of diamond SGFET sensors. The C–F surface decreases its pH sensitivity compared with C–H, C–O, and C–N diamond surfaces [12]. Various types of C–F diamond fabrication methods have been presented, i.e., exposure to dissociated xenon difluoride (XeF_2_) [23,24] or inductively coupled plasma (ICP) treatment with a fluorocarbon gas (C_x_F_y_ gas) source [12]. However, in addition to the well-known issue in which ICP with C_x_F_y_ gas could cause damage to the quality of the crystal structure and surface smoothness [7], the fluorination process of ICP–C_x_F_y_ can form an unexpected fluorohydrocarbon film (C_x_F_y_ film) on the diamond surface, where it could influence its field-effect transistor current–voltage (FET-IV) characteristics if used as a semiconductor chemical sensor.

Here, to overcome the above-mentioned issues, we propose a “fluorine gas direct treatment method” utilizing fluorine gas (F_2_ gas) for the fabrication of a no-gate-insulator diamond SGFET sensor with a smaller amount of unexpectedly generated fluorocarbon film. X-ray photoelectron spectroscopy (XPS) and time-of-flight secondary ion mass spectrometry (TOF-SIMS) were used to investigate the state of the fluorohydrocarbon film on the surface of the C–F diamond, and the current–voltage (IV) characteristics of the diamond FETs created by the fluorine gas direct treatment were also evaluated.

## 2. Materials and Methods

### 2.1. Fabrication of C–F Diamonds

Highly (110)-oriented polycrystalline diamonds that were synthesized by chemical vapor deposition (CVD) were used in this study. The typical specification of the (110) diamond is described in our previous study [25]. Before use, the (110) diamonds were cleaned with ultrapure water, ethanol, acetone, and isopropyl alcohol [25,26]. To obtain a nearly full C–H diamond surface that could be used for fluorination, a hydrogen termination was conducted with an “Applied Science and Technology (ASTeX)-type” microwave plasma chemical vapor deposition system (Brighton, MI, USA).

**Figure 1:**
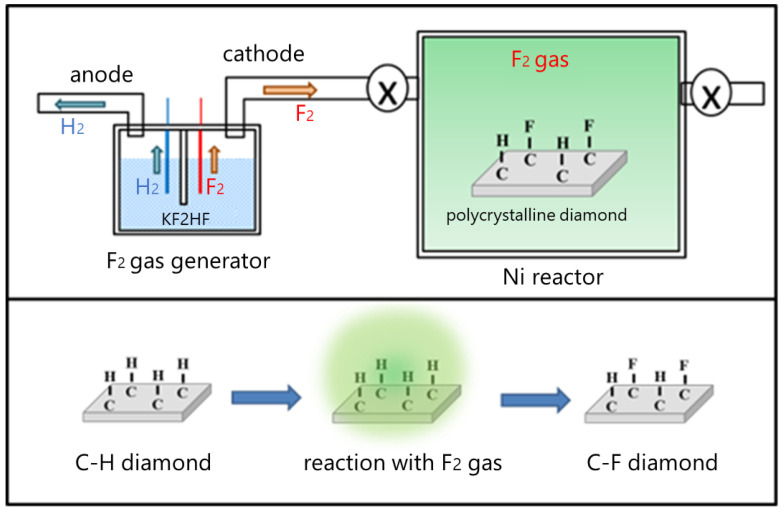
Schematic diagram of the process of fluorine gas (F_2_ gas) direct treatment.

To obtain the C–F diamond surface with a smaller amount of unexpectedly generated C_x_F_y_ film, a F_2_ gas treatment was introduced, where some of the experiments were conducted in collaboration with the F-Technical Assistance Center, TAKAMATSU TEISAN Corporation. Figure 1 shows a schematic diagram of the process of the F_2_ gas treatment method. The process was carried out by using a lab-made nickel (Ni) reactor or a F_2_ reactor. The nearly full C–H diamond substrates were set in the reactor and then the pressure in the reactor was reduced after a purge of argon gas. After decompression, F_2_ gas that was generated by electrolysis of potassium diacid fluoride (KF_2_HF) melted at ca. 100 degrees Celsius (degrees C) was introduced into the Ni reactor. After the F_2_ treatment, the reactor was cooled down to room temperature, and then F_2_ gas in the reactor was purged out with argon gas. The fluorine pressure for the reaction was ca. 101 kPa, and the reaction time was from 30 min to 24 h.

For comparison, conventional ICP treatment with perfluoropropane gas (C_3_F_8_ gas) was applied for the fabrication of C–F diamonds under pressure of 3 Pa and the gas flow of 20 standard cubic centimeters per minute (sccm) with 100–500 W for up to 30 s. To characterize the C–F diamond surface as an SGFET sensor, a boron-doped diamond (BDD) SGFET was fabricated in the same process described in our previous works [12,20].

### 2.2. Characterization of C–F Diamond Surface and Diamond SGFET

A Model 3300 X-ray photoemission spectroscopy (XPS; ULVAC-Phi) device was used for the identification of chemical bonds (C–F, C–O, C–N) on the diamond surface. The functional groups on the diamond were also analyzed by using a Model TRIFT III TOF–SIMS (ULVAC PHI) device. The typical conditions were as follows: the primary ion source was gallium ions; the ion irradiation time was 300 s; the impact energy was 15 kV; the measured mass range was 0.1–200; the mass resolution was 500 at *m*/*z*; and the lateral resolution was 22 μm. Curve fitting of XPS spectra was performed with the analysis software Origin.

The C–F BDD SGFETs were characterized using a measurement system comprising two sets of Model 2400 source-measure units (Keithley Instruments, Inc., Cleveland, OH, USA) and/or two sets of Model GS820 source-measure units (Yokogawa Electric Corp., Tokyo, Japan) for the gate-source voltage (Vgs) and the application of the drain-source voltage (Vds) with common-source mode. Labview 2010 (National Instruments Inc., Austin, TX, USA) was used to control the process and obtain the I–V data. The gate-source voltage was applied via a silver–silver chloride gate electrode (Ag/AgCl electrode). SGFET I–V profiles were obtained at room temperature (approximately 20 degrees C). All chemicals used in this study were of reagent grade and were obtained from Wako, Kanto Kagaku, Tokyo Kasei, and Horiba. Additionally, 1 mM phosphate-buffered saline buffer solution (PBS; pH 7.4) was used for the evaluation.

## 3. Results

### 3.1. Characterization of the C–F Diamond

**Figure 2:**
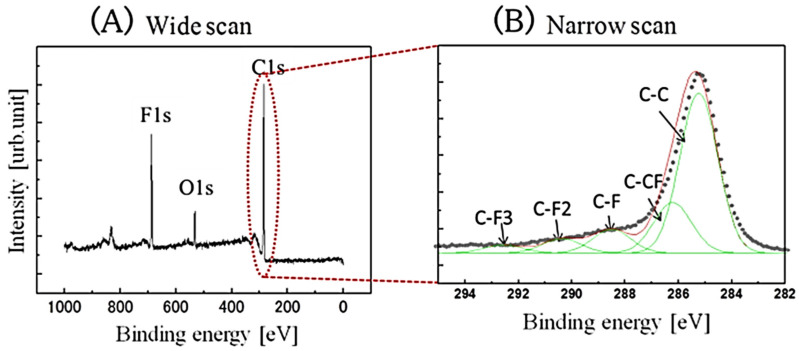
X-ray photoelectron spectra of C–F diamond surfaces fabricated via fluorine gas direct treatment: (**A**) a wide XPS image and (**B**) a narrow scan of C 1s of the C–F diamond surfaces.

**Figure 3:**
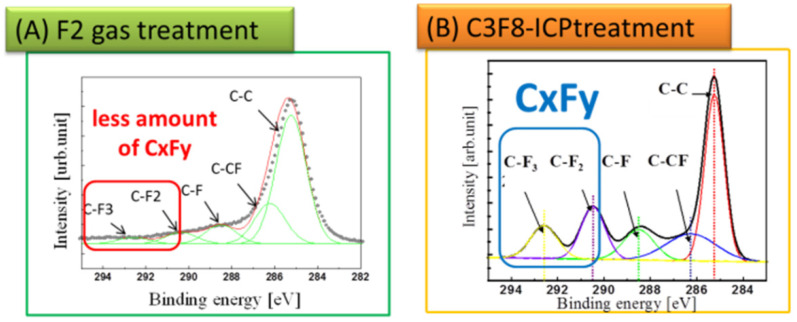
X-ray photoelectron spectra of the C–F diamond surfaces: (**A**) a narrow scan of C 1s of the C–F diamonds fabricated via fluorine gas treatment and (**B**) a narrow scan of C 1s of the C–F diamonds fabricated via perfluoropropane–ICP (C_3_F_8_-ICP) treatment.

**Figure 4:**
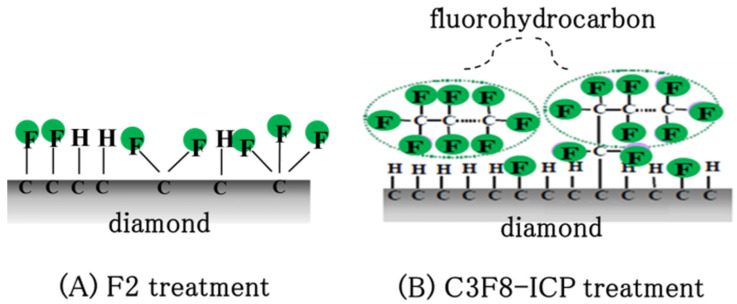
Schematic diagram of the C–F diamond surface: (**A**) a C–F diamond surface with a small amount of fluorohydrocarbon fabricated via fluorine gas treatment (F_2_ treatment) and (**B**) a C–F diamond surface with fluorohydrocarbon fabricated via perfluoropropane–ICP (C_3_F_8_-ICP) treatment.

To evaluate the diamond surfaces after the F_2_ reaction, the fluorine functional groups on the C–F diamond surfaces were analyzed using XPS with a monochromated Al K X-ray source. The coverage of fluorine (F1s; 688 eV) was estimated from the deconvolution of the C 1s peak intensity. The wide spectra in Figure 2A reveal the elemental composition on the C–F diamond films after the direct F_2_ gas functionalization of the surface. The oxygen species existing on the C–F diamond surface may be attributed to physio-adsorbed oxygen after several days of exposure to the air following the C–F treatment. No additional treatment was performed to remove the physio-adsorbed oxygen effect prior to the XPS analysis. The main peak near 284.5 eV is the C 1s peak. The F 1s peak at 688 eV in the spectrum of the F_2_ direct fluorination confirms the surface fluorination of the diamond films. The deconvolutions of the C 1s spectra on the F_2_ direct fluorination are shown in Figure 2B. The deconvolutions can be separated into sp3C (285 eV), C–CF (286.6 eV), C–F (288.8 eV), C–F_2_ (290.9 eV), and C–F_3_ (292.8 eV). The amount of fluorocarbon layers can then be evaluated, in particular, using the amounts of C–F_2_ and C–F_3_ bonds on the diamond surfaces. Figure 3 shows the deconvoluted C 1s of the XPS narrow analysis on the C–F diamonds. Figure 3 shows the C–F diamonds treated by F_2_ gas and C_3_F_8_-ICP. The respective percentages (%) of various types of chemical functionalization derived from the fluorine functional groups on the C–F diamonds were calculated from Figure 3A,B, as summarized in Table 1. In addition, Figure 4 shows a schematic diagram of the proposed surface state of the C–F diamonds, reflecting the XPS analysis results (Figure 3 and Table 1). Because a smaller amount of C–F_3_ was estimated in comparison to C–CF_2_ and C–F on the F_2_-treated C–F diamonds, a fluorocarbon film did not exist or only small amounts existed on the surfaces. However, because the amount of C–F_3_ was nearly the same as that of C–CF_2_ and C–F, a certain amount of fluorocarbon film was present on the surfaces of the C–F diamonds treated by C_3_F_8_-ICP.

**Figure 5:**
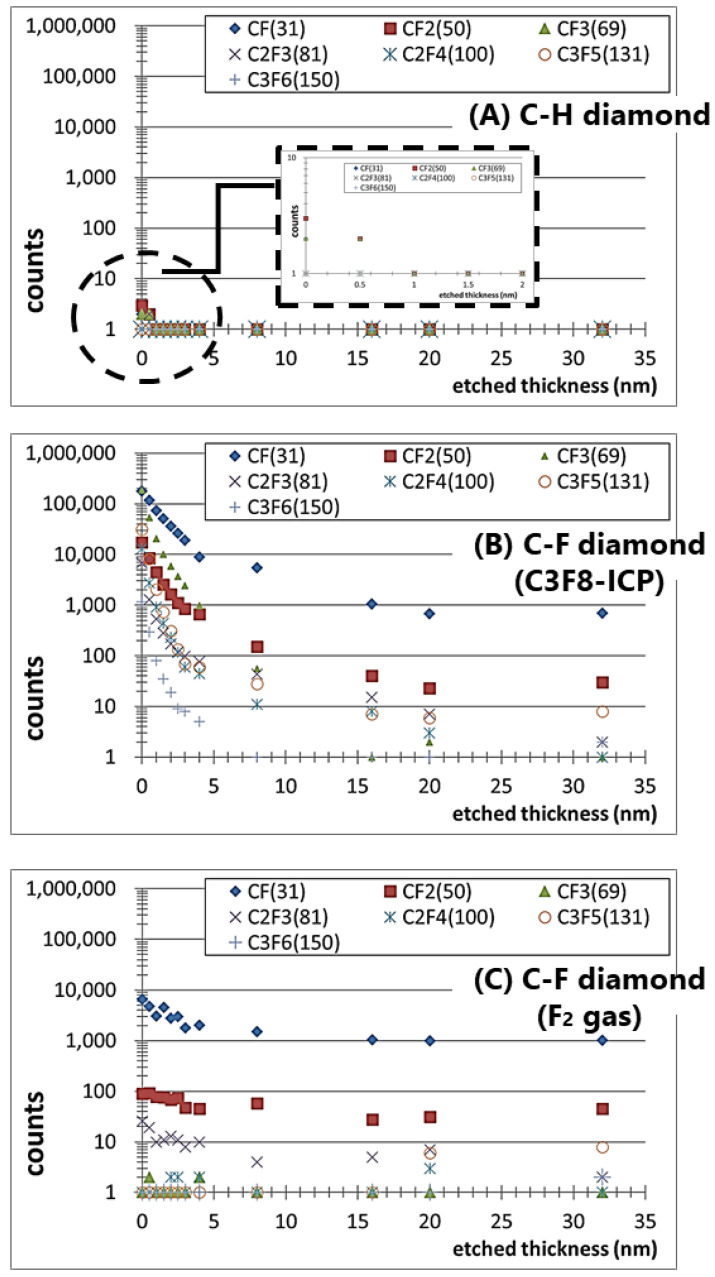
The ratio of fluorine-related fragments as measured by TOF-SIMS analysis: (**A**) C–H diamond; (**B**) perfluoropropane–ICP(C_3_F_8_-ICP)-treated C–F diamond; and (**C**) fluorine-gas(F_2_-gas)-treated C–F diamond.

**Figure 6:**
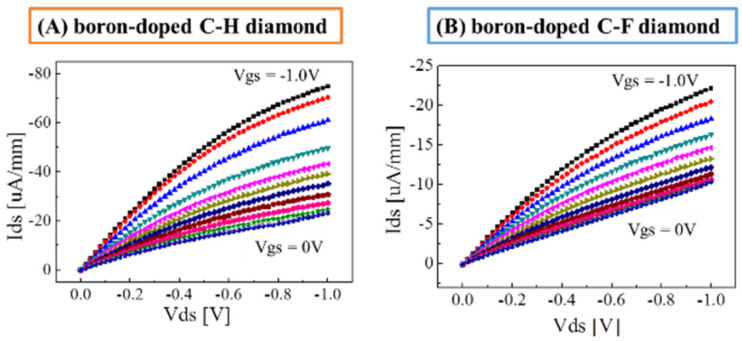
FET Ids–Vds characteristics at Vgs in the range of 0 V to −1.0 V of boron-doped diamond electrolyte-solution-gate FET: (**A**) a C–H diamond SGFET and (**B**) a C–F diamond SGFET fabricated via fluorine gas treatment.

To verify the model in Figure 4, fluorine-related fragments delivered from the fluorine-terminated group and the fluorocarbon films of the C–F diamonds fabricated using F_2_ gas and C_3_F_8_-ICP were analyzed via TOF-SIMS to confirm the presence of fluorohydrocarbons on the C–F diamonds. A C–H diamond was used as a reference sample. Figure 5 shows the detected amount of various fluorocarbon secondary ions in the range of 0–200 amu, where each count was collected under the same conditions (i.e., primary ion dose density, same sample area size, and same beam condition). The fluorocarbon-related fragment peaks are CF + (*m*/*z* 31), CF_2_ + (*m*/*z* 50), CF_3_ + (*m*/*z* 69), C_2_F_3_ + (*m*/*z* 81), C_2_F_4_ + (*m*/*z* 100), C_3_F_5_ + (*m*/*z* 131), and C_3_F_6_ + (*m*/*z* 150). Even though the fluorocarbon-related fragments are nearly undetectable in the case of the C–H diamond (Figure 5A), the C–F diamonds have fluorocarbon-related fragments on the surface and at depth (Figure 5B,C). The detection of CF_3_+ fragments suggests the presence of fluorohydrocarbons on the surfaces of the ICP-treated C–F diamonds. In Figure 6B, the amount of CF_3_+ fragment decreases at greater depths from the substrate surface, and the ratio of fluorine-related fragments becomes essentially constant at the depth where the amount of CF_3_+ fragment is sufficiently reduced. This indicates that fragments derived from the fluorocarbon film are detected on the surface of the substrate but, as the depth increases, fragments derived from the fluorine termination of the diamond substrate are detected. Conversely, Figure 5C shows that the CF_3_+ fragment is not detected on the surface and that the ratio of fluorine-related fragments becomes essentially constant away from the surface. This indicates that a fluorocarbon film does not exist or only small amounts exist on the surfaces of C–F diamonds treated via F_2_ gas reaction.

### 3.2. Characterization of C–F BDD SGFET

For a feasibility study of C–F BDD SGFET as a chemical sensor, the FET current–voltage (FET I–V) characterization of the C–F BDD SGFET was examined with respect to the C–H BDD SGFET. Initially, the SGFETs and an Ag–AgCl gate electrode (the reference electrode) were washed with ultrapure water and then immersed in a 1 M PBS buffer solution (pH 7.4) for 30 min before their FET I–V characteristics were evaluated using a common-source method via drain-source current (Ids)–gate-source (Vgs) characteristics (the so-called static characteristics). Figure 6 shows typical characteristics of the drain-source voltage (Vds) and drain-source current (Ids) of the developed SGFETs. Figure 6A,B show the static characteristics of the C–H BDD SGFET and the C–F BDD SFGET fabricated via F_2_ gas direct fluorination, respectively. The Vgs applied through the Ag–AgCl gate electrode was varied stepwise from −1.0 V to 0 V in steps of 100 mV. For each value of Vgs, the Ids was obtained as a function of the Vds from 0 V to −1.0 V. The drain-source current was pinched off, and the saturation regions and the distinct linear pattern were obtained. Under the condition of Vds of −1.0 V, the Ids of the C–H diamond is −22 μA/mm, whereas the Ids of the fluorine-gas-treated C–F diamond is −11 μA/mm at Vgs of 0 V. This represents a 50% decrease. Under the same condition of −1.0 V Vds, the Ids of the C–H diamond was −78 μA/mm at Vgs of −1.0 V, whereas the Ids of the fluorine-gas-treated C–F diamond was −24 μA/mm at Vds of −1.0 V. A/mm, resulting in a 30% decrease.

Even though the Vth (threshold voltage) shifted by 0.8 V in the negative direction due to the partial fluorine termination, nearly ideal Vds–Ids characteristics according to the metal–oxide–silicon semiconductor field-effect transistor (MOSFET) theory, which describes the Ids as being proportional to the square of the difference between Vgs and Vth, Ids ∞ (Vgs − Vth)^2^, were obtained for both the C–F BDD SGFET and the C–H BDD SGFET. The 0.8 V shift was preserved at larger Vgs, as shown in Figure 6B, indicating that ideal SGFET performance was maintained even with partial fluorine termination.

## 4. Conclusions

In this study, a fluorination treatment method for diamonds and a fabrication method for C–F BDD SGFET with a smaller amount of unexpected fluorocarbon film via the introduction of a F_2_ gas direct reaction were proposed. In comparison to conventional ICP fluorination, the F_2_ gas efficiently reacts with the C–H bond and a fluorocarbon film does not exist or only exists in small amounts on the surface. In addition to a further study of the ion sensitivity of C–F BDD SGFET, long-term characteristics, in addition to short-term characteristics, and the interference of cations/anions/chemicals are to be examined from an application perspective.

## Figures and Tables

**Table 1:** Quantification of the bonding state of fluorine functional groups on the C–F diamond surface.

Bonding Site	Binding Energy (eV)	Coverage (%)	
		F_2_-Treated	C_3_F_8_-ICP-Treated
C–F_3_	292.8	2.2	10.8
C–F_2_	290.9	5.5	17.1
C–F	288.8	9.9	9.7
C–CF	286.6	19.9	8.6
C–C(sp3)	285	62.4	53.9

## Data Availability

Data sharing is not applicable to this article.

## References

[1] Tinwala H., Wairkar S. (2019). Production, surface modification and biomedical applications of nanodiamonds: A sparkling tool for theranostic. Mater. Sci. Eng. C.

[2] Kondo T., Ito H., Kusakabe K., Ohkawa K., Einaga Y., Fujishima A., Kawai T. (2007). Plasma etching treatment for surface modification of boron-doped diamond electrodes. Electrochim. Acta..

[3] Shiomi H. (1997). Surface damages in diamond by Ar/O_2_ plasma and their effect on the electrical and electrochemical characteristics of boron-doped layers. Jpn. J. Appl. Phys..

[4] Yagi K., Tsunozaki D., Tryk A., Fujishima A. (1999). Control of the Dynamics of Photogenerated Carriers at the Boron-Doped Diamond/Electrolyte Interface by Variation of the Surface Termination. Electrochem. Solid-State Lett..

[5] Baumann P.K., Nemanich R.J. (1998). Surface cleaning, electronic states and electron affinity of diamond (100), (111) and (110) surfaces. Surf. Sci..

[6] Ramesham R. (1998). Effect of annealing and hydrogen plasma treatment on the voltammetric and impedance behavior of the diamond electrode. Thin Solid Films.

[7] Bobrov K., Mayne A., Comtet G., Dujardin G., Hellner L., Hoffman A. (2003). Atomic-scale visualization and surface electronic structure of the hydrogenated diamond C(100)-(2x1):H surface. Phys. Rev. B.

[8] Kondo T., Ito H., Kusakabe K., Ohkawa K., Honda K., Einaga Y., Fujishima A., Kawai T. (2008). Characterization and electrochemical properties of CF4 plasma-treated boron-doped diamond surfaces. Diam. Relat. Mater..

[9] Martin H.B., Argoitia A., Angus J.C., Landau U. (1999). Voltammetry Studies of Single-Crystal and Polycrystalline Diamond Electrodes. J. Electrochem. Soc..

[10] Durrant S.F., Peterlevitz A.C., Castro S.G., Landers R., Bica de Moraes M.A. (2001). Characterization of diamond fluorinated by glow discharge plasma treatment. Diam. Relat. Mater..

[11] Ferro S., Battisti A.D. (2003). The 5-V Window of Polarizability of Fluorinated Diamond Electrodes in Aqueous Solutions. Anal. Chem..

[12] Shintani Y., Kobayashi M., Kawarada H. (2017). An All-Solid-State pH Sensor Employing Fluorine-Terminated Polycrystalline Boron-Doped Diamond as a pH-Insensitive Solution-Gate Field-Effect Transistor. Sensors.

[13] Ferro S., Battisti A.D. (2003). Physicochemical Properties of Fluorinated Diamond Electrodes. J. Phys. Chem. B.

[14] Luo D., Ma D., Liu S., Nakata K., Fujishima A., Wu L. (2022). Electrochemical reduction of CO_2_ on fluorine-modified boron-doped diamond electrode. Diam. Relat. Mater..

[15] Siné G., Ouattara L., Panizza M., Comninellis C. (2003). Electrochemical Behavior of Fluorinated Boron-Doped Diamond. Electrochem. Solid-State Lett..

[16] Kawarada H., Araki Y., Sakai T., Ogawa T., Umezawa H. (2001). Electrolyte-Solution-Gate FETs Using Diamond Surface for Biocompatible Ion Sensors. Phys. Status Solidi..

[17] Edgington R., Ruslinda A.R., Sato S., Ishiyama Y., Tsuge K., Ono T., Kawarada H., Jackman R.B. (2012). Boron δ-doped (111) diamond solution gate field effect transistors. Biosens. Bioelectron..

[18] Edgington R., Sato S., Ishiyama Y., Morris R., Jackman R.B., Kawarada H. (2012). Growth and electrical characterisation of δ-doped boron layers on (111) diamond surfaces. J. Appl. Phys..

[19] Sasaki Y., Kawarada H. (2010). Low drift and small hysteresis characteristics of diamond electrolyte-solution-gate FET. J. Phys. D Appl. Phys..

[20] Shintani Y., Ibori S., Igarashi K., Naramura T., Inaba M., Kawarada H. (2016). Polycrystalline boron-doped diamond with an oxygen-terminated surface channel as an electrolyte-solution-gate field-effect transistor for pH sensing. Electrochim. Acta.

[21] Shintani Y., Ibori S., Kawarada H. (2019). Deoxyribonucleic-acid-sensitive Polycrystalline Diamond Solution-gate Field-effect Transistor with a Carboxyl-terminated Boron-doped Channel. Anal. Sci..

[22] Falina S., Kawai S., Oi N., Yamano H., Kageura T., Suaebah E., Inaba M., Shintani Y., Syamsul M., Kawarada H. (2018). Role of Carboxyl and Amine Termination on a Boron-Doped Diamond Solution Gate Field Effect Transistor (SGFET) for pH Sensing. Sensors.

[23] Rietwyk K.J., Wong S.L., Cao L., O’Donnell K.M., Ley L., Wee A.T.S., Pakes C.I. (2013). Work function and electron affinity of the fluorine-terminated (100) diamond surface. Appl. Phys. Lett..

[24] Foord J.S., Singh N.K., Jackman R.B., Gutierrez-Sosa A., Proffitt S., Holt K.B. (2001). Reactions of xenon difluoride and atomic hydrogen at chemical vapour deposited diamond surfaces. Surf. Sci..

[25] Syamsul M., Kitabayashi Y., Matsumura D., Saito T., Shintani Y., Kawarada H. (2016). High voltage breakdown (1.8 kV) of hydrogenated black diamond field effect transistor. Appl. Phys. Lett..

[26] Shintani Y., Kawarada H. (2017). Polycrystalline Boron-doped diamond electrolyte-solution-gate field-effect transistor for an ap-plication to the measurement of water percentage in ethanol. Anal. Sci..

